# Three *Leptospira* Strains From Western Indian Ocean Wildlife Show Highly Distinct Virulence Phenotypes Through Hamster Experimental Infection

**DOI:** 10.3389/fmicb.2019.00382

**Published:** 2019-03-11

**Authors:** Colette Cordonin, Magali Turpin, Jean-Loup Bascands, Koussay Dellagi, Patrick Mavingui, Pablo Tortosa, Marjolaine Roche

**Affiliations:** ^1^ Unité Mixte de Recherche Processus Infectieux en Milieu Insulaire Tropical (UMR PIMIT), Université de La Réunion, INSERM 1187, CNRS 9192, IRD 249, Plateforme de Recherche CYROI, Sainte-Clotilde, Reunion, France; ^2^ Unité Mixte de Recherche Diabète Athérothrombose Thérapie Réunion-Océan Indien (UMR DéTROI), Université de La Réunion, INSERM U1188, Plateforme de Recherche CYROI, Sainte-Clotilde, Reunion, France

**Keywords:** *Leptospira*, leptospirosis, virulence, hamster model, kidney, bats, tenrecs, Western Indian Ocean

## Abstract

Leptospirosis is one of the most widespread zoonoses worldwide, with highest incidence reported on tropical islands. Recent investigations carried out in a One-Health framework have revealed a wide diversity of pathogenic *Leptospira* lineages on the different islands of Western Indian Ocean carried out by a large diversity of mammal reservoirs, including domestic and wild fauna. Using golden Syrian hamsters as a model of acute infection, we studied the virulence of *Leptospira interrogans, L. mayottensis,* and *L. borgpetersenii* isolates obtained from rats, tenrecs, and bats, respectively. Hamsters were inoculated with 2.10^8^ bacterial cells and monitored for 1 month. The *L. interrogans* isolate proved to be the most pathogenic while *L. mayottensis* and *L. borgpetersenii* isolates induced no clinical symptoms in the infected hamsters. High leptospiral DNA amounts were also detected in the urine and organs of hamsters infected with the *L. interrogans* isolate while *L. mayottensis* and *L. borgpetersenii* isolates mostly failed to disseminate into the organism. In addition, histological damage was more pronounced in the kidneys and lungs of hamsters infected with the *L. interrogans* isolate. Altogether, these data support that *Leptospira* strains shed by mammals endemic to this insular ecosystem (*L. mayottensis* and *L. borgpetersenii* isolates) are less pathogenic than the *L. interrogans* rat-borne isolate. These results may provide a relevant framework for understanding the contrasting epidemiology of human leptospirosis observed among Western Indian Ocean islands.

## Introduction

Leptospirosis is a neglected re-emerging bacterial zoonosis affecting over one million persons annually and causing nearly 60,000 deaths worldwide (Costa et al., 2015). This disease is caused by a spirochete from the genus *Leptospira* (Adler, 2015) and human infection most commonly results from indirect environmental contamination with leptospires shed by the urine of infected reservoir animals. In humans, asymptomatic infection is more common than the symptomatic disease which may be associated with mild symptoms such as fever, headache, vomiting, or more severe symptoms including pulmonary hemorrhage, liver and renal failure, also known as Weil’s syndrome (Evangelista and Coburn, 2010).

Leptospirosis can reach high incidence in human populations especially in the tropics where humid and warm climate can promote survival of the bacteria (Simbizi et al., 2016). The tropical islands of Western Indian Ocean report some of the highest incidence worldwide (Pappas et al., 2008; Derne et al., 2011). Despite their importance, unfortunately little is known about the precise nature of pathogenic strains in this region, partly due to the fact that very few have been successfully isolated from either human clinical cases or animal hosts. Rats are well-known reservoirs of leptospirosis worldwide, including Western Indian Ocean islands, but they are not the only mammals at play. For example, dogs and cattle were also identified as potential reservoirs for the disease on Reunion while *Tenrec ecaudatus*, introduced from Madagascar to Mayotte as game meat, may play a role in human leptospirosis (Guernier et al., 2016; Lagadec et al., 2016). In Madagascar, a wide range of endemic mammals, including several tenrec and bat species, has been reported to shelter a wide diversity of *Leptospira* lineages although there is no evidence of their role as disseminators of the disease (Lagadec et al., 2012; Dietrich et al., 2014; Gomard et al., 2016; Guernier et al., 2016).

Recently, molecular studies have described contrasting epidemiology of leptospirosis between the islands of Western Indian Ocean. On Reunion Island, most cases of leptospirosis are caused by *Leptospira interrogans* with sequence types (STs) also distributed in other countries worldwide (Guernier et al., 2016). By contrast, studies conducted on human cases from Mayotte reported a variety of original STs belonging to *L. interrogans*, *L. kirschneri*, *L. borgpetersenii,* and *L. mayottensis* species (Bourhy et al., 2012, 2014). In addition, severity of human leptospirosis is significantly different on the two islands with 4.6% admission in intensive care unit and 0.7% mortality rate in Mayotte while Reunion records 30% admission in intensive care unit and 5% mortality (Cire Océan Indien, 2017; Pagès et al., 2017; Subiros et al., 2017).

In this study we aim to provide a first insight into differences in virulence between *Leptospira* strains from Western Indian Ocean islands shed by different mammal hosts through experimental infection of golden Syrian hamsters. This rodent model mimics the clinical symptoms of human leptospirosis and has already allowed the description of several virulence phenotypes of different *Leptospira* strains (Tucunduva de Faria et al., 2007; Silva et al., 2008; Villanueva et al., 2014; Matsui et al., 2015). To pursue our goal, we tested three available *Leptospira* isolates obtained from wild fauna during a previous research program. The *L. interrogans* isolate was obtained from a rat on Reunion and displays a ST associated with the majority of acute human cases on Reunion Island (Guernier et al., 2016) and the *L. mayottensis* isolate obtained from a tenrec on Mayotte has a ST previously reported in several human acute cases on the island (Lagadec et al., 2016). By contrast, the *L. borgpetersenii* isolate was obtained from a bat endemic to Madagascar and has a genotype that has not been reported from any human case thus far.

## Materials and Methods

### Animal Experiments and Ethical Statement

Experiments were conducted following the guidelines of the Office Laboratory of Animal Care at the CYROI platform. Female golden Syrian hamsters (Janvier Labs, Le Genest, France), aged from 6 to 8 weeks, were housed by 3 in enriched cages. The appropriate number of animals was calculated using the Resource equation method (Festing, 2006). All animal procedures carried out in this study were performed in accordance with the European Union legislation for the protection of animals used for scientific purposes (Directive 2010/63/EU). Experimental procedures were approved by the French Ministry of Sciences and Higher Education under the number APAFIS#8773-2016111615105111 v2.

### Bacterial Isolates

*Leptospira* strains used in this study were isolated from the kidneys of animals trapped in the field. *L. interrogans* was isolated on Reunion Island from *Rattus rattus* (2013RR GLM983) (Guernier et al., 2016), *L. mayottensis* on Mayotte from *Tenrec ecaudatus* (2014TE MDI222) (Lagadec et al., 2016) and *L. borgpetersenii* on Madagascar from *Triaenops menamena* (2014TM FMNH228863), an insectivorous bat endemic to Madagascar (Lebarbenchon et al., 2017). Leptospires were grown at 28°C for 14–70 days in Ellinghausen-McCullough-Johnson-Harris (EMJH) liquid medium (Difco, Detroit, MI, USA) supplemented with albumin fatty acid supplement (AFAS) and 5-fluorouracil (5-Fu) as previously described (Lagadec et al., 2016; Biscornet et al., 2017). Of note, all used isolates were passaged less than ten times *in vitro* before restoration of virulence.

### Restoration of Virulence

As the virulence of pathogenic *Leptospira* tends to attenuate during *in vitro* passages and freeze storage (Reed et al., 2000; Samir and Wasfy, 2013), the assessment of virulence requires a restoration step in hamsters by serial infections (Haake, 2006). For this, two serial passages in hamsters were performed and the resulting isolates were subsequently used for experimental infection. Cultures were centrifuged at 9,000 rpm for 30 min, pellets resuspended in 500-μl sterile phosphate buffer saline (PBS) 1X, and bacterial cells counted using an improved Neubauer cell counting chamber (Marienfeld Superior, Germany). Animals were inoculated intraperitoneally (i.p.) with 1.5–3 × 10^8^ leptospires in 500 μl of sterile PBS 1X and sacrificed 3–7 days post-infection (dpi). Urine and kidney samples were used to inoculate EMJH liquid medium supplemented with AFAS and 200 μg/ml 5-Fu as previously detailed,^1^ followed by a subculture in the same fresh medium without 5-Fu. Each strain was subcultured less than 5 times *in vitro* in order to maintain the virulence of leptospires used for experimental infection (Reed et al., 2000).

### Infection and Sample Collection

Hamsters were inoculated i.p. with 2 × 10^8^ low-passaged *L. interrogans* (*n* = 9 animals), *L. mayottensis* (*n* = 7), or *L. borgpetersenii* (*n* = 7) resuspended in 500 μl of sterile PBS 1X. Control animals (*n* = 7) were injected i.p. with 500 μl of sterile PBS 1X. Animals were monitored for 4 weeks. Urine samples were collected from each hamster once a day and subsequently frozen at −80°C. After 4 weeks or earlier when moribund (characterized by significant weight loss, lethargy, isolation, and ruffled fur), hamsters were anesthetized with 5 mg/kg xylazine and 25 mg/kg ketamine injected i.p. and subsequently euthanized by cardiac puncture. The lungs, liver, spleen, kidneys, and brain were collected and processed for further experiments or immediately frozen at −80°C.

### Bacterial Load Measurement

Up to 25 mg of frozen kidney, lung, liver, and brain, and up to 10 mg of frozen spleen tissue were collected and lysed in ATL buffer (Qiagen, Germany) for at least 3 h. DNA was extracted from frozen organs and from up to 100 μl of frozen urine samples using the DNEasy Blood & Tissue kit (Qiagen, Germany). DNA amounts were quantified using a probe-specific real-time PCR (Smythe et al., 2002) and a Quantinova probe PCR mix (Qiagen, Germany). Each sample was triplicated and considered positive if at least two out of the three triplicates led to positive PCR with Ct < 45. For organ samples, results are expressed as mean genome copies per milligram of tissue ± standard error of mean (SEM), determined using data of all hamsters within a group. DNA amounts in urine were determined for each animal and are reported as genome copies per microliter of urine. The number of genome copies was calculated considering the genome size of *L. interrogans* strain Fiocruz L1–130 (Nascimento et al., 2004).

### Viability of Leptospires Assay

A portion of fresh liver, kidney, or brain was used to inoculate fresh EMJH medium as previously detailed^2^. Cultures were observed once a week under a dark-field microscope (Axio Lab.A1, Zeiss France) and considered negative if no leptospires were visible after 4 months.

### Histological Studies

A portion of fresh lung and one whole kidney were fixed in 4% paraformaldehyde for 24–48 h, dehydrated in five successive ethanol baths (70, 90, 95, 2 × 100%, 1 h each) and two successive xylene baths, 1 h each. Dehydrated organs were incubated in two successive melted paraffin baths for 1 h and overnight, respectively, and eventually in paraffin. Four- to six-μm-thick paraffin sections of kidney were deparaffinized in three successive xylene baths, 10 min each, and rehydrated in three successive ethanol baths (100, 70, and 50%, 2 min each) and one bath of distilled water, 2 min. Rehydrated sections were stained with either periodic acid Schiff or Sirius Red and sections of lung were stained with hematoxylin and eosin. Stained sections were observed with a digital slide scanner (NanoZoomer S60, Hamamatsu France).

### Statistical Analysis

Log-rank test was used to compare survival curves of the different groups of hamsters. Non-parametric Kruskal-Wallis followed by non-parametric Dunn’s multiple comparison tests were used to compare mean body weight percentage and *Leptospira* DNA amounts in organs of hamsters. Statistical analyses were conducted using RStudio 1.0.153 (RStudio Team, 2015).

## Results

### Body Weight Monitoring, Clinical Signs, and Survival Analysis

**Figure 1:**
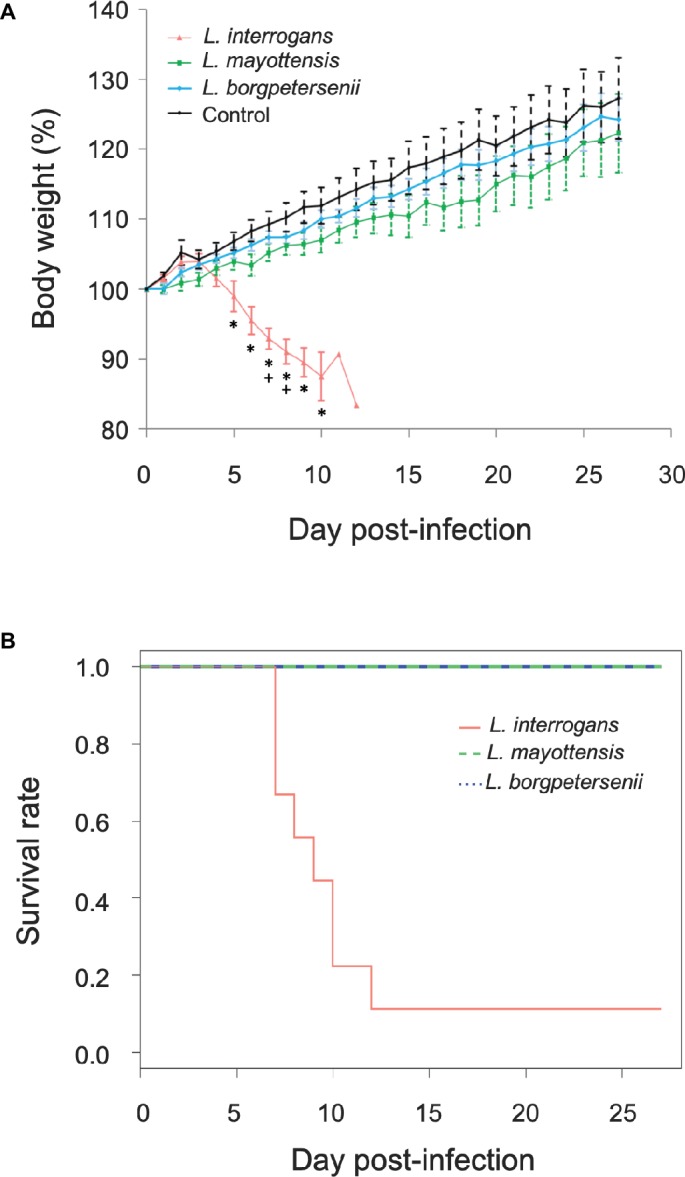
Hamsters monitoring. **(A)** Growth curves of control hamsters (black, *n* = 7) and hamsters infected with *L. interrogans* (red, *n* = 8), *L. mayottensis* (green, *n* = 7), or *L. borgpetersenii* (blue, *n* = 7). Results are expressed as means ± SEM. Asterisks (*) indicate significant differences in body weight percentage between hamsters infected by *L. interrogans* and control hamsters. Plus signs (**+**) indicate significant differences in body weight percentage between hamsters infected with *L. interrogans* and two other groups of hamsters: control and *L. borgpetersenii*-infected hamsters. The hamster surviving the infection with *L. interrogans* was considered as an outlier and was not included in the growth curve data. **(B)** Survival rates of hamsters infected with *L. interrogans* (red, *n* = 9), *L. mayottensis* (green, *n* = 7), or *L. borgpetersenii* (blue, *n* = 7).

Groups of hamsters were inoculated i.p. with 2 × 10^8^ bacteria and subsequently monitored for 4 weeks. None of the hamsters infected with *L. borgpetersenii* and *L. mayottensis* exhibited any clinical sign and all challenged animals survived throughout the whole experiment with a mean weight increase of 24 and 22%, respectively, comparable with the weight increase measured on control hamsters (27%) (Figure 1A). By contrast, the survival rate of hamsters infected with *L. interrogans* dropped drastically from 7 days after the infection compared to hamsters infected with *L. mayottensis* and *L. borgpetersenii* (*p* = 0.0019) (Figure 1B): only one out of the nine *L. interrogans-*infected hamsters actually survived during the 4 weeks of experiment without any clinical sign and a weight increase of 23%. Moribund *L. interrogans*-infected hamsters showed weight loss averaging 7.08% in the first week, and reaching 16.65% between 7 and 12 dpi (Figure 1A). Other clinical signs included ruffled fur, lethargy, and isolation.

### Dynamics of *Leptospira* Urinary Shedding

**Figure 2:**
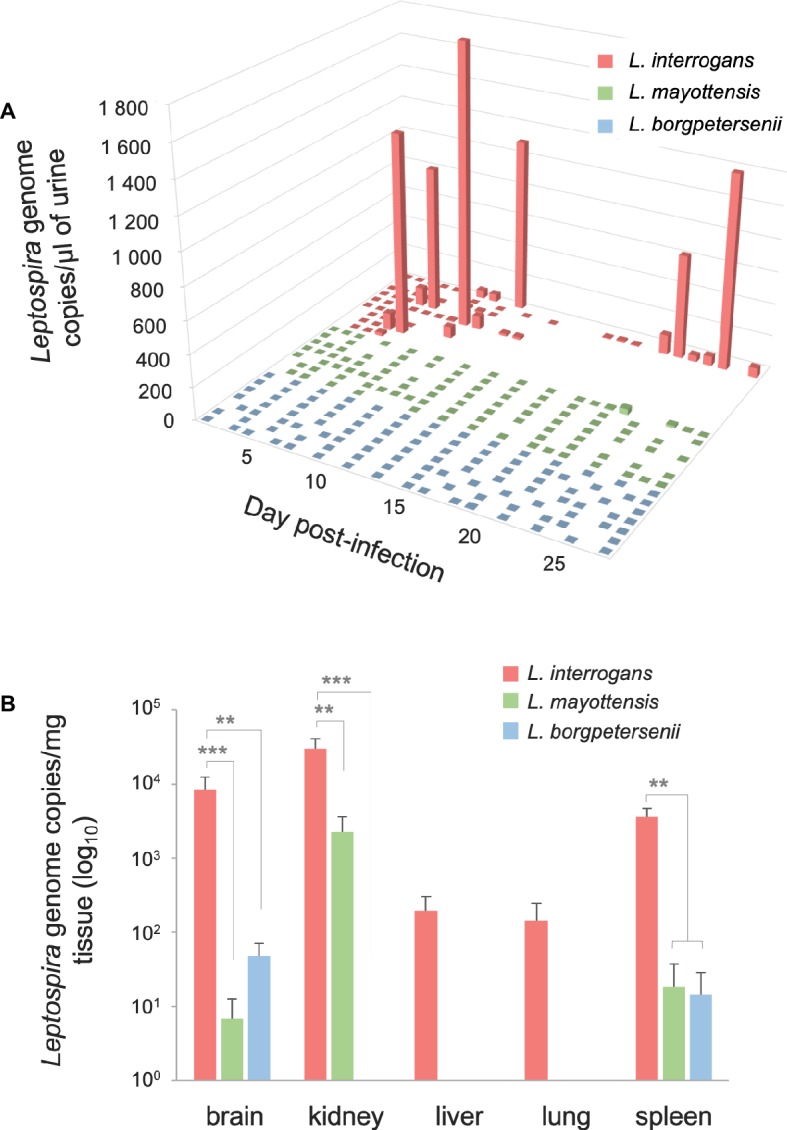
Quantification of *Leptospira* DNA in urine and organs. **(A)** Pathogenic *Leptospira* urinary shedding in hamsters infected with *L. interrogans* (red, *n* = 9), *L. mayottensis* (green, *n* = 7), or *L. borgpetersenii* (blue, *n* = 7). Individual data are presented for each hamster. **(B)** Hamsters infected with *L. interrogans* are represented in red (*n* = 8), *L. mayottensis* in green (*n* = 7), and *L. borgpetersenii* in blue (*n* = 7). DNA amounts in organs were calculated at the time of death for *L. interrogans*-infected hamsters and after 4 weeks of experiment for *L. mayottensis*- and *L. borgpetersenii*-infected hamsters. Results are expressed as means ± SEM. Differences in DNA amount were tested with non-parametric Kruskal-Wallis test followed by Dunn’s multiple comparison test. After Dunn’s test, a *p*-value below 0.025 indicated a significant difference; ** *p* < 0.01; *** *p* < 0.001. The hamster surviving *L. interrogans* infection was considered as an outlier and was not included in the DNA amounts data.

The urine of hamsters was collected once a day whenever possible in order to follow the dynamics of *Leptospira* shedding following infection (Figure 2A). *Leptospira* DNA was detected discontinuously in the urine of all *L. interrogans-*infected hamsters: high peaks of *Leptospira* DNA, ranging from 820.62 (±159.79) to 1839.19 (±414.13) genome copies per microliter of urine, were detected in the urine of five out of nine hamsters as early as 5 dpi. Among these animals, the single hamster surviving *L. interrogans* infection excreted high amounts of *Leptospira* DNA throughout the 4 weeks of infection. The other four *L. interrogans*-infected animals showed limited leptospiral excretion during the whole experiment (<80 *Leptospira* genome copies/μl). By contrast, hamsters infected with *L. borgpetersenii* and *L. mayottensis* isolates shed little or no *Leptospira* DNA in their urine (up to 2.54 and 35.72 genome copies/μl, respectively).

### Quantification of *Leptospira* DNA in Organs

*Leptospira* DNA was quantified in brain, kidney, liver, lung, and spleen tissues of euthanized animals. As shown on Table 1, highest DNA amounts were obtained in kidney tissues, the highest amount being detected in the single hamster surviving *L. interrogans* infection (1.7 × 10^6^ ± 2.9 × 10^5^ genome copies/mg). *Leptospira* DNA amounts were higher in *L. interrogans*-infected animals for all tested tissues: DNA amount was increased by over 10-fold in the kidneys and over 100-fold in the brain, lungs, and spleen tissues of animals infected with *L. interrogans* as compared to those infected with *L. mayottensis* or *L. borgpetersenii* (Figure 2B). *Leptospira* DNA was detected in the liver of *L. interrogans*-infected animals only. All kidney tissues from *L. interrogans*-infected hamsters tested positive through PCR, while only three out of seven *L. mayottensis*- and none of the *L. borgpetersenii*-infected animals tested positive using DNA from their kidney tissues (Table 1). DNA was detected at low levels (< 40 genome copies/mg) in the spleen and brain of hamster infected with either *L. mayottensis* or *L. borgpetersenii* (Figure 2B).

**Table 1:** Rate of *Leptospira* positive samples using qPCR or culture on organs of experimentally infected hamsters.

	Positive qPCRs/Tested animals (%)					Positive cultures/Tested animals (%)		
	Brain	Kidney	Liver	Lung	Spleen	Brain	Kidney	Liver
*L. interrogans*	8/9 (88.9)	9/9 (100.0)	4/9 (44.4)	3/9 (33.3)	7/8 (87.5)^*^	8/9 (88.9)	8/9 (88.9)	3/9 (33.3)
*L. mayottensis*	2/7 (28.6)	4/7 (57.1)	0/7 (0.0)	1/7 (14.3)	1/7 (14.3)	0/7 (0.0)	2/7 (28.6)	0/7 (0.0)
*L. borgpetersenii*	3/7 (42.9)	0/7 (0.0)	0/7 (0.0)	0/7 (0.0)	1/7 (14.3)	5/7 (71.4)^#^	2/7 (28.6)^#^	0/7 (0.0)

* Data missing for one hamster.

# The isolates were lost after one passage in vitro.

Data indicate number of positive qPCRs and cultures at the time of death for L. interrogans-infected animals and at 4 weeks post-infection for L. mayottensis- and L. borgpetersenii-infected hamsters.

### Viability of Leptospires

Considering previous detection of high levels of *Leptospira* DNA in the kidneys and liver of infected hamsters (Coutinho et al., 2014; Wunder et al., 2016), possible brain damage induced by the disease (Panicker et al., 2001; Chang et al., 2003), and culture constraints, we selected these three organs for detection of live leptospires through culture (Table 1). Live leptospires were obtained from almost all kidney and brain homogenate cultures and in one-third of liver homogenate cultures from *L. interrogans-*infected hamsters. Intriguingly, no leptospires were seen in any tissue culture prepared from the single surviving *L. interrogans-*infected hamster. Organs from *L. mayottensis*-infected hamsters led to lower isolation success with no positive brain or liver homogenate cultures and only 2 out of 7 positive kidney homogenate cultures. Although lost after one passage *in vitro*, live leptospires were obtained from animals infected with *L. borgpetersenii* mainly using brain tissue and to a lesser extent using kidney tissue (Table 1).

### Differential Tissue Damages Revealed by Histological Monitoring

**Figure 3:**
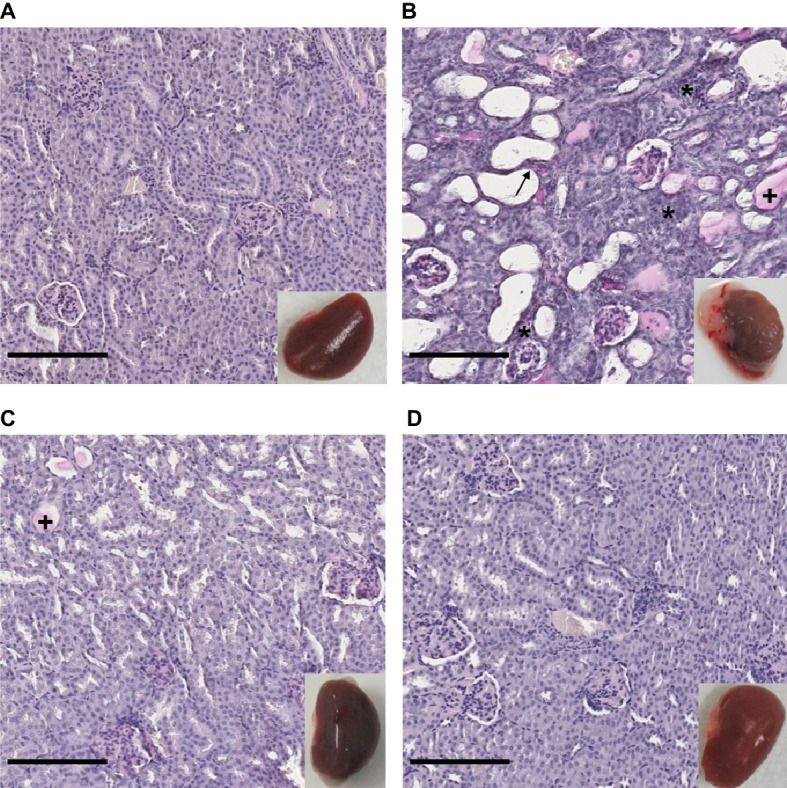
Kidney structure from control and infected hamsters. Microscopic observations of kidney sections stained with periodic acid Schiff obtained from control animals **(A)** and animals infected with *L. interrogans* **(B)**, *L. mayottensis* **(C)**, or *L. borgpetersenii* **(D)** 4 weeks after infection. Atrophic tubules (arrow), hyaline casts (+), and cellular infiltrates (*) are indicated. Scale bars: 150 μm. Macroscopic observations of kidneys are shown in inserts.

The renal tropism of pathogenic *Leptospira* and the pulmonary involvement in most severe human forms of leptospirosis (Vijayachari et al., 2008) led us to consider kidneys and lungs for histological studies. Gross external examination of organs showed significant lesions in the kidneys of hamsters infected with *L. interrogans* (Figure 3, see lower right inserts). Lungs of *L. interrogans-* and *L. mayottensis-*infected hamsters harbored some lesions while the lungs of *L. borgpetersenii*-infected hamsters showed a normal phenotype, indistinguishable from control lungs (data not shown).

**Figure 4:**
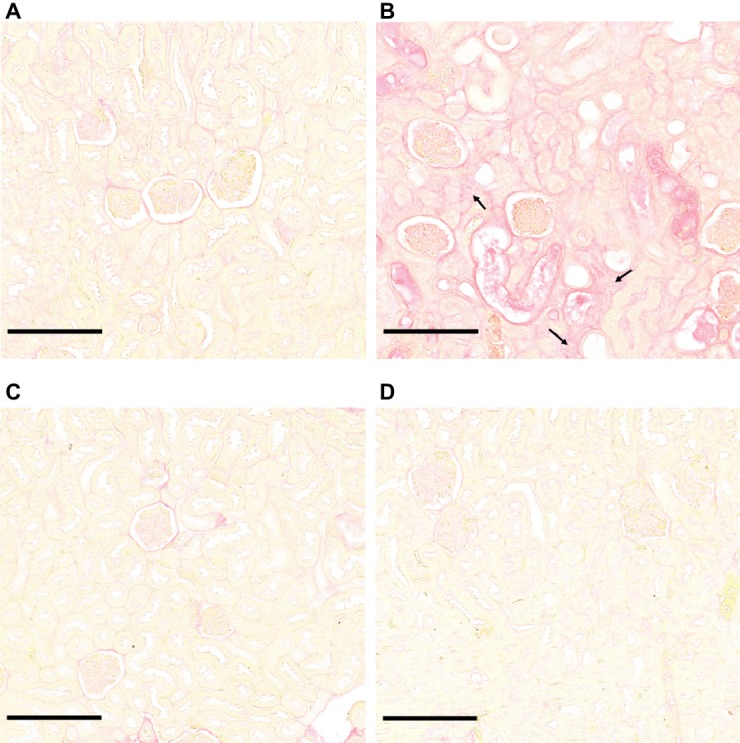
*L. interrogans*-induced interstitial fibrosis. Microscopic observations of kidney sections stained with Sirius Red obtained from control animals **(A)** and animals infected with *L. interrogans* **(B)**, *L. mayottensis,* **(C)** or *L. borgpetersenii* **(D)** 4 weeks after infection. Arrows indicate areas of interstitial fibrosis. Scale bars: 150 μm.

Microscopic sections of kidneys from control hamsters and from hamsters infected with *L. borgpetersenii* stained with periodic acid Schiff showed glomeruli and tubules of regular shape 4 weeks after infection. Slightly dilated tubules and some hyaline casts were visible in kidney sections from *L. mayottensis*-infected hamsters. Kidneys from hamsters infected with *L. interrogans* showed retracted glomeruli with large urinary space, focal infiltrates of inflammatory cells mainly around vessels, and very important tubular damages characterized by tubular dilation, atrophy, and hyaline casts (Figure 3). Sirius Red staining further highlighted interstitial fibrosis in kidneys from these animals (Figure 4).

**Figure 5:**
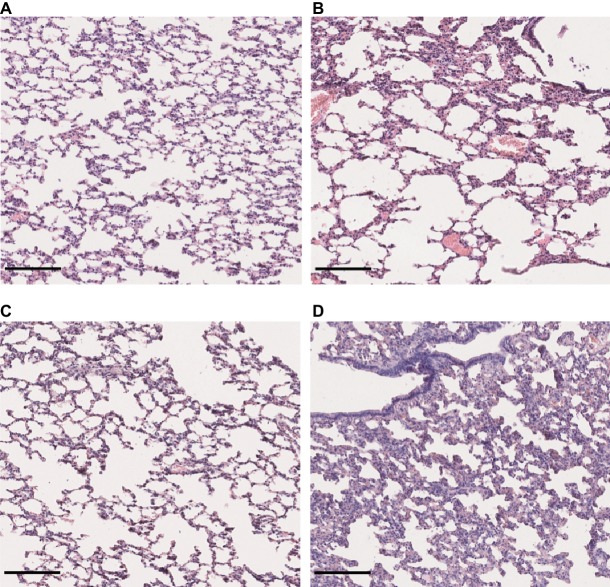
Lung structure from control and infected hamsters. Microscopic observations of lung sections stained with hematoxylin and eosin obtained from control animals **(A)** and animals infected with *L. interrogans* **(B)**, *L. mayottensis,* **(C)** or *L. borgpetersenii* **(D)** 4 weeks after infection. Scale bars = 150 μm.

Lung sections stained with hematoxylin and eosin revealed a mild to moderate thickening of alveolar septa in the lungs of hamsters infected with *L. interrogans* and *L. borgpetersenii*, at the time of death and after 4 weeks of infection, respectively. Dilated alveoli were also observed in the lungs of *L. interrogans*-infected hamsters. By contrast, lungs of control and *L. mayottensis*-infected hamsters euthanized after 4 weeks showed a regular structure (Figure 5).

**Figure 6:**
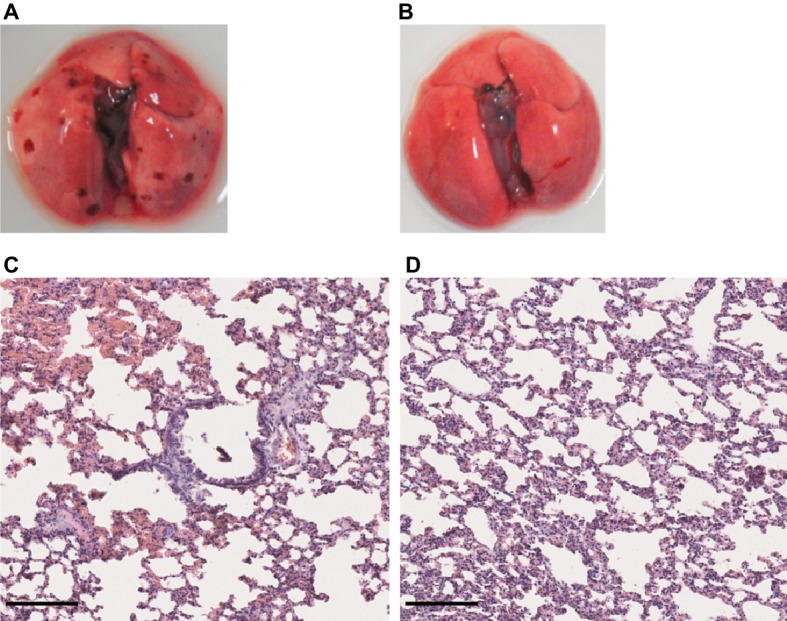
Lung structure from *L. mayottensis-*infected hamsters sacrificed at intermediate timepoints. Macroscopic examination of lungs **(A,B)** and lung sections stained with hematoxylin and eosin **(C,D)** from *L. mayottensis*-infected hamsters sacrificed at 10 **(A,C)** or 17 dpi **(B,D)**. Scale bars = 150 μm.

Given that no clinical sign was observed in the first week of infection in hamsters infected with either *L. mayottensis* or *L. borgpetersenii*, we chose to sacrifice only one hamster from each group at two different intermediate timepoints, 10 and 17 (*L. mayottensis* group) or 18 (*L. borgpetersenii* group) dpi, in order to explore possible internal damages. At 10 dpi, the hamster infected with *L. mayottensis* harbored several petechial hemorrhages in the lungs and histological analysis revealed focal areas of hemorrhage and thickening of alveolar septa. At 17 dpi, no macroscopic lesion but thickening of alveolar septa were observed in the lungs; no area of hemorrhage was observed (Figure 6). Lungs of *L. borgpetersenii*-infected hamsters sacrificed at 18 dpi harbored the same features as at the end of the experiment (data not shown).

## Discussion

Western Indian Ocean islands harbor a large diversity of pathogenic *Leptospira* lineages carried out by a wide range of animal reservoirs (Dietrich et al., 2018). In this study, we selected three regional *Leptospira* strains and evaluated their virulence phenotype using golden Syrian hamsters as a model of acute disease. The obtained data show that *L. mayottensis* 2014TE MDI222 and *L. borgpetersenii* 2014TM FMNH228863 isolates endemic to Madagascar and surrounding islands are both less virulent than *L. interrogans* 2013RR GLM983 isolate from Reunion. All but one hamster infected with *L. interrogans* isolate showed significant weight loss and had to be euthanized a few days after the challenge, in keeping with previous experimental infections (Forster et al., 2013; Villanueva et al., 2014). It was either difficult or impossible to collect urine from these hamsters, likely as a result of kidney failure, which was further confirmed by histological examinations that showed tissue damages. Unexpectedly, no areas of intensive hemorrhage were observed in the lungs of challenged animals, which differs from previous studies reporting foci of intensive hemorrhage in *L. interrogans*-infected hamsters (Nally et al., 2004; Villanueva et al., 2014). The absence of profuse pulmonary lesions could result from the high infectious dose used in our experiments: the hamsters could have died before the infection invaded the lungs (Diniz et al., 2011). As for the single surviving hamster, a recent study has shown that females are more resistant than males to *L. interrogans* infection (Gomes et al., 2018). The obtained low survival rate might be actually zero if males had been used instead of females. Interestingly, as previously reported (Haake, 2006), qPCR showed that this single survivor supported persistent renal infection.

By contrast, *L. mayottensis* and *L. borgpetersenii* isolates failed to induce clinical symptoms on the infected hamsters. Intriguingly, necropsy at 4 weeks revealed macroscopic pulmonary lesions in some *L. mayottensis*-hamsters but not in *L. borgpetersenii*-hamsters although histological analysis revealed a thickening of alveolar septa in the latter. The absence of early symptoms in these two groups led us to euthanize animals at intermediate timepoints in order to address possible tissue damage in the course of the experiment. No damage was observed at 10 and 18 dpi in *L. borgpetersenii*-infected hamsters. *L. mayottensis*-infected hamsters showed pulmonary damage at 10 dpi but not at 17 dpi. Histological findings confirmed these observations with the presence of focal hemorrhage and alveolar septal thickening at 10 dpi and only alveolar septal thickening at 17 dpi. This pattern is suggestive of an attempt of lung repair, a phenomenon previously described in hamsters experimentally infected with *L. interrogans* (Marinho et al., 2009). The confirmation of such pattern will require the implementation of an experimental setup allowing to monitor the dynamics of infection and tissue lesions induced by *L. mayottensis*.

Leptospiral tissue tropism following experimental infection differed among bacterial isolates. The high *L. interrogans* DNA amounts and the presence of live leptospires in the brain and kidneys of infected hamsters suggest that these organs are preferential targets of *L. interrogans*. Retracted glomeruli, dilated tubules, and an abundance of hyaline casts in the kidneys have commonly been observed in *L. interrogans* infection (Arean, 1962; Zhang et al., 2012; Forster et al., 2013). Although the induction of nervous system damage by *L. interrogans* has been previously reported in human cases (Forwell et al., 1984; Brown et al., 2003; Chang et al., 2003; El Bouazzaoui et al., 2011), experimental infection studies mainly report damage and high leptospiral load in the kidneys, lungs, and liver only (Silva et al., 2008; Lourdault et al., 2009; Fujita et al., 2015). Four weeks following infection, *L. mayottensis* was present only in the kidneys as indicated by high leptospiral DNA amounts and the presence of live leptospires. However, at 10 dpi the presence of live leptospires in cultures inoculated with kidney, brain, and liver homogenates indicates that *L. mayottensis* could actually disseminate into several organs in the early phases of infection.

*L. borgpetersenii* isolate was found to be the least virulent strain used in this study. This isolate induced no clinical sign and very low leptospiral DNA amounts were detected in the different organs. Leptospires were observed in some cultures from brain and kidney homogenates albeit with low density and with bacteria being lost after one passage *in vitro*. The low *Leptospira* DNA amounts and the absence of cultivable leptospires in the organs from hamsters sacrificed at intermediate timepoints are suggestive of poor dissemination although observation of alveolar thickening indicates that the lungs can be affected by this *Leptospira* lineage. This differs from infection patterns observed with other *L. borgpetersenii* strains inducing important systemic damage and death in hamsters (Villanueva et al., 2014; Matsui et al., 2015). A previous study comparing the virulence of two *L. borgpetersenii* strains, both serovar Hardjo, reported that the avirulent strain was able to efficiently colonize the kidneys of hamsters even at a lower dose (Zuerner et al., 2012). Hence, the bat-borne *Leptospira* used herein clearly behaves differently. Although genotyping defines this isolate as *L. borgpetersenii*, Malagasy bats shelter a huge diversity of unique *L. borgpetersenii* lineages (Gomard et al., 2016) and a complete genome sequencing of this particular isolate is required to determine its exact phylogenetic position.

We can hypothesize that these distinct virulence phenotypes may partly explain the contrasted epidemiology of human leptospirosis in Western Indian Ocean region. Indeed, MLST profile of *L. interrogans* 2013RR GLM983 isolate, found in other countries worldwide, is the same as that of *L. interrogans* identified in most human leptospirosis acute cases on Reunion while *L. mayottensis* 2014TE MDI222 isolate, considered as endemic to Western Indian Ocean, has the same MLST profile as *L. mayottensis* identified in clinical cases on Mayotte. By contrast, *L. borgpetersenii* 2014TM FMNH228863 isolate, which induced no clinical sign in hamsters, has not yet been identified in human cases of leptospirosis. On Reunion, a narrow diversity of mainly *L. interrogans* is involved in clinical cases while a wider diversity of *Leptospira*, distributed in four distinct species, is identified in clinical cases on Mayotte. On Reunion, mostly introduced mammals like rats, mice, or dogs, were identified as *Leptospira* carriers (Guernier et al., 2016), while on Mayotte, endemic small mammals like tenrecs may also have epidemiological importance in human leptospirosis (Lagadec et al., 2016). The French islands of Mayotte and Reunion hence provide an interesting environmental setup, as transmission chains are distinct on both islands and associated with human infection of contrasting severity. The understanding of such epidemiological contrast could be addressed through a prospective study to be conducted on Mayotte in which the symptoms of each human case are described together with each associated *Leptospira* lineage/species. This will allow addressing whether one species or lineage is responsible for most of the fatal cases, and testing whether the remaining endemic lineages may be associated with milder symptoms.

In conclusion, our data suggest that the *L. interrogans* 2013RR GLM983 isolate is significantly more virulent than both *L. mayottensis* 2014TE MDI222 and *L. borgpetersenii* 2014TM FMNH228863 isolates, endemic to Western Indian Ocean. Golden Syrian hamsters provide a good experimental model for exploring virulence features of *Leptospira* strains found in Western Indian Ocean and the contrasted epidemiology of leptospirosis observed in the islands of this region. Furthermore, presented data pave the way for future investigations aiming at illuminating genetic determinants and mechanisms of virulence.

It has been previously reported that the regulation of genes encoding cytokines can differ according to the *Leptospira* strain used to infect hamsters (Fujita et al., 2015). This differential regulation of cytokine expression has also been observed among human patients. More specifically, the up-regulation of cytokines was more important in patients developing a severe disease compared to patients with mild disease (Reis et al., 2013). The inflammatory response may also have an involvement in the development of the disease. A previous study reported an absence of leptospires in the lungs of infected hamsters although pulmonary damage was observed (Tomizawa et al., 2017). It would be interesting to compare the inflammatory response induced by the three isolates used in this study and determine the impact of the potentially different regulation on the phenotype of infected hamsters.

The determinants of virulence are poorly known in *Leptospira* (Lehmann et al., 2014; Buyuktimkin et al., 2015; Patra et al., 2015; Fouts et al., 2016), notably because genetic tools are scarce, hence impairing reverse genetics approaches and because the basal position of spirochetes is associated with a high number of proteins of unknown function. The unique diversity of pathogenic *Leptospira* sheltered by mammals mostly endemic to Western Indian Ocean region provides a biological material that is suitable for the investigation of virulence determinants through genome-wide approaches such as comparative genomics.

## Author Contributions

CC, KD, PT, MR, and PM conceived and designed the experiments. CC, MR, PT, MT, and J-LB performed the experiments. CC, PT, PM, MR, and J-LB contributed resources, reagents, materials, and analysis tools. CC wrote the paper. All co-authors revised the paper.

### Conflict of Interest Statement

The authors declare that the research was conducted in the absence of any commercial or financial relationships that could be construed as a potential conflict of interest.

## Footnotes

^1^http://dx.doi.org/10.17504/protocols.io.ifccbiw

^2^http://dx.doi.org/10.17504/protocols.io.ifccbiw
